# On the Curative Effects of Loss of Blood

**Published:** 1830-10-01

**Authors:** 


### On the Curative Effects of Loss of Blood. By *Marshall Hall,* M.D. &c. &c.

[Second and concluding Article.]

The talented author of the work before us has so mingled or mystified, we had almost said, original matters with the substance of preceding publications, that it would require much more time than we are able to spare, to separate the new from the old. Let us take the first passage of the introduction to this division of the volume under review, and our readers will probably be quite familiar with the words.

"It is one of the most remarkable facts in physic, that if several patients of similar strength and constitution, but affected by dissimilar diseases, be respectively placed in the erect position and bled to deliquium, they will be found to have lost very various quantities of blood. I have known a patient not apparently very feeble, faint on losing four ounces of blood; and I have known patients bear to lose fifty, sixty, and even seventy ounces of blood without syncope." 175.

Our readers well remember that the foregoing passage forms part of an avant-courier, which Dr. H. sent forth some time ago,* with the view of collecting information on the effects of blood-letting. A long extract from a paper published by Mr. Heming in the Medical Gazette, concludes the first section of this chapter.

Dr. Hall then proceeds to particularize certain specific diseases in their relation to loss of blood, beginning very naturally with fever.

### I. FEVER.

The different forms and complications of this and of inflammation, are of very frequent occurrence; and, in their intimate nature and relation to blood-letting, "pure fever and pure inflammation are widely different."

"There have been long disputes, indeed, whether fever ever be perfectly pure, that is, independent of inflammation. In a practical point of view, I think it the safest plan to regard fever as occasionally truly idiopathic, but as extremely liable to be conjoined with inflammation.

Whoever is imbued with an accurate knowledge of physiology will, I think, perceive in the phenomena of fever, much that is to be distinctly referred to the state of the nervous system: the alternate chills and heat, the tendency to vertigo, the muscular tremors, the affection of the various secretions, are plainly of this character. With this, the vascular system soon participates. They form a whole which suffers together.

Fever seems to differ from inflammation in being an affection of the whole nervous and vascular systems; in inflammation there is an affection of these systems in one part or organ.

There is another difference between these two diseases: fever appears to consist in an affection of the nervous system and of the heart and larger arteries, the capillary vessels being only affected as an extension of this morbid state. In inflammation there is, according to the experiments of Dr. Wilson Philip, and Dr. Hastings, a primary affection of the capillary vessels, consisting in enlargement of their diameter and a slower movement of more numerous globules of the blood. A consequence which flows from this view of the subject is, that to subdue momentarily the state of fever, we have only to subdue the augmented action of the heart and larger arteries; but as the capillary circulation is less immediately under the influence of the heart, the action of the former may be subdued, whilst a morbid state of the latter may be continued with comparatively little change.

It is upon this principle, I believe, that a fact is to be explained which will be frequently adverted to in this work, that syncope is more readily produced by the abstraction of blood, in pure fever, and in other diseases consisting alike in the state of the heart and larger arteries, than in pure inflammation, consisting in a peculiar condition of the capillary vessels, more permanent and less under the influence of the general circulation.

In the former case, syncope is the simple effect of depriving the heart and arteries of their accustomed stimulus, and this probably under circumstances of augmented susceptibility of the nervous system to impressions of this kind; in the latter, although blood may be taken, and the action of the heart and arteries be thus subdued, yet from a less degree of susceptibility of the nervous system, and from the unsubdued morbid action of the capillaries, acting as it were as a

* See No. XXIII. of this Journal, page 40, et seq.

permanent stimulus to the general system, syncope is not so soon induced by the abstraction of blood. But whatever the explanation of this fact may be, the fact itself is, I think, established upon the sure ground of multiplied experiment." 199.

There are three circumstances, Dr. H. observes, in fever, which should lead to the use of the lancet—excessive reaction of the vascular system—much excitement of the nervous system—local inflammation. On each of these cases our author expends a few remarks. In excessive vascular reaction, he observes, bleeding is of essential service, especially when employed early—the limit, beyond which it would be dangerous to go, being clearly marked out by the degree of susceptibility to the effects of loss of blood, denoted by the tendency to syncope on abstracting blood pretty freely in the erect posture.

"The same observation may be made in regard to great nervous excitement denoted by delirium. To abstract a moderate quantity of blood does great good. But to bleed too freely is dangerously to depress the powers of life. In this case, as in the last, the patient may safely be placed in the erect posture, and bled to incipient syncope, if it be a first bloodletting and early in the disease. But the most marked difference in regard to the powers of supporting the loss of blood, is superinduced by the addition of a local inflammatory affection to the original disease. The patient immediately becomes less prone to faint on being bled. It will be obvious how important it would be to establish this point accurately by an ample collection of facts; and thus to trace it in its reference to practice. It appears to me, from what I have hitherto ascertained, that there is, in every instance, a strict alliance between the degree of tolerance of loss of blood and the exigencies of the cure." 201.

II. *Inflammation*. Dr. Hall's remarks on this point are very brief. He would observe that inflammation is not necessarily ushered in by rigor, or attended by heat of skin, except in very severe cases. In the slighter instances he thinks the rigor generally depends upon a superadded cause, existing in the state of the bowels—while the heat depends upon this cause, or upon the use of remedies. He has also observed that the pulse, in pure inflammation, is very little accelerated at first; but is so in the severest cases. In pure inflammation, except of the encephalon itself, the head (he observes) is frequently little affected, there being neither head-ache nor vertigo—though these symptoms eventually come on, together with delirium, "as the result of depletion and exhaustion."

"But the remark of greatest moment which I would make in this place, is, that pure inflammation induces a state of the system which protects it from the influence and effects of loss of blood. A patient under the influence of pure inflammation, will bear to lose a far greater quantity of blood without experiencing syncope, than the same person in health. This fact is of the utmost interest and importance in a practical point of view." 203.

Inflammation he considers as a "sort of concentrated stimulus, exciting and maintaining the powers of the system." We should rather say that it is an *irritation* producing violent and *irregular* action in the system. Thus while the action of the vascular apparatus is excited in inflammation, the functions of various organs, especially the digestive, are almost annulled. We agree with our author, however, that, under the influence of inflammation, blood-letting is well borne. The protective influence of inflammation differs from that of fever. "In the latter, syncope subdues all the actions;—in the former, those peculiar to the inflammation still subsist, being, in a great measure, independent of the action of the heart." This fact, according to our author's experience, obtains in a more marked manner, in inflammation of the encephalon than in that of any other organ of the body. It is also more observable in inflammation of the serous membranes, and parenchymatous structures, than in that of the mucous membranes. The following case is put forth to illustrate, first, the protecting powers of inflammation in regard to the effects of loss of blood—and, in the second place, these very effects, when this peculiar influence is withdrawn.

"Mrs. ———, aged 30, and of moderate strength, had suffered from pain in the right hypochondriac region for a month, when on the evening of the 14th of May, 1828, it became extremely acute.

On the morning of the 15th, it continued very severe, interrupting the respiration, and preventing the patient from assuming the recumbent position; the pulse was 90, full, and hard; the surface hot. She was placed quite erect, and bled to syncope; this took place when thirty-nine ounces of blood had flowed, the pain being relieved but not entirely removed. Calomel and purgative medicines were given. In the evening the pain had increased, the pulse remained as in the morning, the medicines had acted well; Mrs. ——— was again placed in the erect position and bled to syncope, and the same quantity of blood flowed as before; the pain being quite removed. A blister was applied, and the medicine repeated with colchicum.

On the 16th, the pain continued relieved; the countenance was pallid; the pulse less frequent; the skin cooler.

On the 19th, there was no pain of the side, the countenance was pallid, but apt to flush frequently; the pulse was 96, and throbbing; with much throbbing of the head, ringing of the ears, and complaint of weakness.

On the 20th, Mrs. ——— was found subject to flushing, palpitation of the heart, and frightful dreams; the pulse was 90, but without throbbing; the respiration unattended by pain; but there was still some degree of tenderness on pressure in the right hypochondrium." 208.

In another case the most active blood-bletting had been employed to subdue peritonitis; but an abscess formed and burst into the bowels. At this moment the most acute symptoms of re-action from loss of blood were set up, and the patient suffered from the most violent throbbing pain of the head, intolerance of sound, palpitation, &c.

"In a third case detailed to me by Dr. Abercrombie, a similar tumor formed on the right side of the pelvis, the inflammation of this tumor greatly subsided by the use of the ordinary remedies; but the patient became affected with delirium. Such cases having been fatal under other treatment, Dr. Abercrombie prescribed a glass of wine to be given every hour. After the fourth glass the patient was found composed. The tumor eventually suppurated externally, and the patient did well." 208.

Dr. Hall does not recommend blood-letting to be carried to actual syncope, "but only to the very first signs of approaching syncope, which is, in fact, to be prevented by immediately laying the patient in the recumbent position." He thinks that, in most of the cases in which much reaction has followed syncope, there has either been no inflammation, or it was subdued completely by the bleeding.

"But there is another remark of the utmost moment, which I must make though cursorily, in this place, as it will require a more particular notice hereafter. It is, that at the very moment the protective influence of inflammation is withdrawn, further bloodletting is in the highest degree dangerous. I have known several instances of the fatal issue of bloodletting when this measure has been instituted as a preventive against the recurrence of symptoms of inflammation which had been subdued by previous bloodlettings.

If I were to venture to state the average quantities of blood which would flow in the different cases and forms of inflammation, I should mention forty ounces for arachnitis, from thirty to thirty-five for pleuritis or pneumonia, and fifteen for bronchitis. This simple statement cannot fail to strike the medical reader. It is impossible to foresee at once all the advantages which must flow in practice, from these important differences in the powers or susceptibilities of the system in regard to bloodletting in these different diseases.

I think it important to mention, in a very especial manner, that in some forms of acute anasarca, there is great tolerance of loss of blood." 210.

### III. IRRITATION.

Dr. H. next proceeds to notice this morbid affection, of frequent occurrence, "and with which the profession generally appear to me still to be totally unacquainted." This statement, Dr. H. thinks, will not be deemed too strong, if he is enabled to shew that "there is a series of cases, not generally distinguished from certain inflammation, and yet very different in their nature, and especially in their reference to the effects of loss of blood." Granting the existence of this series of cases to the fullest extent, it would not prove that the profession generally is "totally unacquainted" with the subject of irritation. We are ready to grant, however, that irritation is very frequently mistaken for inflammation by the routine practitioner, and that much mischief is daily done by this mistake. But, in truth, it is not always very easy, even for the most skilful and attentive practitioner to discriminate in these cases, and where the practitioner is in doubt, he naturally concludes that it is safest to err on the safe side, and run the risk of treating irritation for inflammation, rather than the reverse. Yet there is scarcely less mischief done by the one mistake than by the other.

"The cases to which I allude, resemble, in their symptoms, the most acute forms of arachnitis, pleuritis, and peritonitis, but especially arachnitis. Yet instead of possessing the power of resisting the effects of loss of blood belonging to inflammation, there is the utmost degree of susceptibility to those effects. *In the former cases thirty, forty, and even fifty ounces of blood may flow before the slightest deliquium is observed; in the latter there is frequently the most perfect syncope on abstracting nine or ten ounces of blood!*

The irritation of a calculus in the ureter, or in the hepatic duct, is well known to occasion a remarkable sympathetic affection of the stomach. The introduction of a bougie into the urethra sometimes induces rigor and a complete paroxysm of fever. Uterine irritation is not less frequently the cause of extraordinary effects upon the system generally and upon various organs.

But of all the sources of sympathetic morbid affections, irritation in the stomach and bowels appears to be the most common, and certainly not the least important. Indigestible substances taken, and disordered fæculent matter retained, are the frequent sources of that combined affection of the head and the stomach, termed sick headache." 212.

If such effects of local irritation upon distant organs be admitted, our author thinks it cannot be considered extraordinary that others less recognized should exist. The most frequent cause of this affection, says Dr. H. is a disordered state of the colon—the next is, " some indigestible substance taken into the stomach." These generally require some superadded cause before the morbid affection can be produced. Some shock sustained, or some effort made by the system, is necessary to rouse into activity the cause of irritation otherwise dormant. In the same manner, indigestible substances may frequently be taken, when the health is unimpaired, with impunity; but if the system be under the influence of a shock, or effort, or of nervous or vascular excitement or exhaustion, a cause of disorder which might otherwise lie inert, proves of frightful activity.

" The effects of intestinal or nervous irritation are chilliness, varying from coldness of the extremities to extreme rigor, followed by great heat of the surface, and symptoms resembling those of arachnitis or peritonitis, singly or successively, in their most acute forms, but especially arachnitis; more rarely there is pain resembling that of pleuritis; more rarely still, a peculiar pain passing along one side of the neck to the shoulders; and occasionally, generally after blood-letting, there is palpitation of the heart.

It must be regarded as extraordinary that such marked affections have not been discriminated, and traced to their proper source. But I am persuaded that they are, to this day, confounded with inflammation of the organs chiefly affected, to the great injury, and even danger of the patient. It is indeed extraordinary how slow the human mind is to receive new impressions, even of the truth, wedded as it usually is to first opinions.

These observations apply particularly to that form of this affection which resembles arachnitis. There are few who distinguish it from arachnitis itself. I have, however, witnessed some very interesting scenes, and not less interesting convictions of the truth of the views which I have taken of this subject, in cases which have occurred in the persons or in the families of medical gentlemen themselves." 214.

One of the earliest cases which excited our author's attention to these effects of irritation, was that of a delicate married woman, aged 35, who appeared to be labouring under inflammation of the peritoneum, the symptoms of which were so severe, as apparently to demand the lancet and leeches, by which 30 or 40 ounces of blood were abstracted. The bowels were freely purged—the stools fetid. All the symptoms being removed on the third day, a speedy and secure convalescence was expected; but, on the fourth day, Dr. H. was urgently requested to see the patient.

" She had been seized with severe pain of the head, especially over the eyebrows, attended by beating and throbbing, and by the most urgent intolerance of light, so that the eyes could not be opened for a moment for examination; the pain was increased on attempting to sit up erect; the countenance was palish and sallow; the pulse full and frequent; there was no faintness or sighing.

As this case occurred very early in my investigation of the effects of irritation, I hesitated in determining whether the symptoms were such as I had already witnessed in one or two cases as arising from that cause, or were indicative of inflammation within the head. I prescribed a draught with thirty drops of the tinctura opii and of the spiritus ammoniæ aromaticus, and called again in an hour and a half, not without anxiety. I was greatly relieved to find my patient better in every respect, able to bear the light, suffering much less pain, and having enjoyed a comfortable sleep after a night of wakefulness and distress. Aperient medicine was administered, and, after the full evacuation of the bowels, light nourishment, and a repetition of the draught with tinctura opii and spiritus ammoniæ aromaticus, whilst a cold lotion was applied to the head.

On the succeeding day, Mrs. —— was better in every respect, but complained of any noise. On the next day she was comparatively well, only suffering from vertigo on raising the head.

From this time the recovery was progressive and uninterrupted, the utmost care being taken to regulate the bowels and the diet." 217.

The next case related is that of a young man, aged 19, who complained, on the 29th September, of shooting pain through the region of the stomach to the back, recurring at intervals. These symptoms went off till nearly the same hour next day, when he became affected with coldness of the hands and feet, flushing of the face, violent and constant pain of the head, numbness of the right hand, and contraction of the right side of the lip—slight incoherence—intolerance of light and sound. About two hours after this attack, sickness came on—a great load of matters was vomited—and he became more collected, though still complaining of his head. Some aperient medicine was given, and he recovered in a day or two, without any depletion.

"The next patient, an intelligent surgeon, tall and robust, had undergone a painful operation on the anus, and had suffered much for six days on passing the fæces and on dressing the wound; he had kept himself low, and had passed restless nights as well as painful days. On the morning of the sixth day, after some exertion, the feet and legs became extremely cold; the surface afterwards became generally heated and the mouth clammy; the face was flushed, the skin sore, and the eye-balls particularly tender; the pulse rose to 96. In the afternoon the chilliness returned, and was followed by heat, throbbing in the temples, and pain the head, with flying stitches in the side; the pulse was 112. Sixteen ounces of blood were taken from the arm; faintness and perspiration were induced, the throbbing ceased, but the pain continued, and the pulse was 116; the patient felt overcome, became restless, and affected with vertigo on moving, and frequent sighing.

At night the head became distractingly painful; the faintness exceedingly distressing; I found my patient with wet cloths applied over the forehead and eyes. The question of further bloodletting or of the application of leeches was proposed to me. I was persuaded, however, that the affection of the head depended upon intestinal and nervous irritation. I therefore prescribed a large enema of gruel and oil; with sal volatile, and nourishment.

The enema induced vomiting, and a copious alvine evacuation. On the following day all symptoms of affection of the head had subsided.

In this case the natural strength of the patient, the violence, and even urgency, of the symptoms, the recovery without further bloodletting, and the extreme susceptibility to the effects of loss of blood, are all remarkably displayed, and form a striking contrast with the characters of true arachnitis." 220.

The following case, which we shall abridge, is offered as a not less interesting illustration of this morbid affection.

Mrs. ——, aged 24 years, had suffered from an attack similar to the present, some months previously. She was excited by company, and then began to complain of head-ache, sense of fatigue, and unrefreshing slumbers. Two days afterwards, she had alternate chilliness and flushings, the head-ache augmenting. These symptoms became exasperated during the next two days, the coldness of the extremities being accompanied by great pain, succeeded by extreme dryness and intense heat. The tongue was loaded—the stomach sick—the bowels disordered. During the two next days, these symptoms went on increasing—the head-ache being accompanied by intolerance of light and throbbing, unabated by aperient medicine and rest. It was deemed proper to draw blood; and the patient being placed in the perpendicular posture, the stream was allowed to flow till faintness took place. Syncope was produced by the abstraction of 12 ounces of blood. The distressing symptoms continued till next day, when a discharge of scybalous and unhealthy fæces gave permanent relief.

"In both these cases the symptoms of affection of the head persisted after the bloodletting, and were relieved by sickness and a free evacuation of the bowels. In both, the prostration from the bloodletting was extraordinary." 222.

The following case we shall give in our author's own words.

"Mrs. ——, a young married lady, in the fourth month of pregnancy, habitually costive. The present attack came on after much fatigue in travelling; and she is stated to have experienced a similar one formerly.

On the 7th of October, she complained of pain of the head, and leeches were applied to the temples. On the 8th the pain of the head was more violent and attended with much throbbing of the temples; and to these symptoms pain of the right side under the breast, a sense of tightness across the chest, and hurry in breathing, were superadded. Twelve ounces of blood were drawn, and an efficient aperient medicine was given, and on the 9th and 10th she was much better; and a saline medicine was prescribed.

On the 11th she was again taken worse, after imprudently sitting up; the beating of the temples, tightness across the chest, and difficulty in breathing returned, unattended by cough. Sixteen ounces of blood were taken from the arm, with great relief, and the aperient medicine was repeated.

The patient was relieved, and continued better on the 12th. In the night of the 13th the medical attendant received an urgent message to visit his patient, and found her affected with severe pain and beating of the head, great tightness and pain across the chest, and with violent palpitation of the heart. Twelve ounces of blood were taken, and calomel and other aperient medicines given, with considerable relief.

On the 14th a physician was consulted, who prescribed the pil. hydrarg. with an aperient draught. In the night the apothecary was again sent for, all the symptoms having returned, and now, for the first time, with the addition of a slight cough. Eight ounces of blood being drawn, great relief was obtained.

In the night of the 16th the medical attendant was again sent for; all the symptoms had returned in a still more aggravated form, the pain of the head, tightness across the chest, palpitation, and cough being extremely severe. Eight ounces of blood were drawn without relief; the head was shaved, a cold lotion applied, and a blister ordered for the back of the neck.

On the 17th I saw the patient for the first time: there were much pain and throbbing of the head, which felt benumbed and heavy as if she could not raise it from the pillow; there had been no sleep; the pupils were extremely small, with intolerance of noise and disturbance of any kind; there were palpitation of the heart and sometimes faintness and a feeling of sinking or dying; there were a sense of tightness across the chest, oppression in the breathing, and a peculiar tracheal or laryngal cough; some pain in the region of the uterus increased by pressure, but no vaginal discharge;—the countenance was usually pale, but sometimes flushed, the tongue extremely loaded, and even black at the back part; the alvine evacuations, on giving purgative medicine, were still, at first, dark-coloured, offensive, and scybalous, and afterwards, offensive and like yeast; the pulse was 120.

I was forcibly struck by a general but marked resemblance of this case, to those already given, and to others of the same nature which I had witnessed; the depleting plan already fully adopted and repeated had proved ineffectual in affording lasting relief; the purgatives hitherto given, were, I believed, inefficient. The plan I proposed was, to give efficient purgatives, to restrain their operation by draughts with tinctura opii and spiritus ammoniæ aromaticus, to support the strength by means of nourishment given every hour or oftener, to procure sleep by anodyne enemata, to guard against exertion or fatigue, noise or disturbance. The recovery was uniformly progressive; there was not even one recurrence of the painful attacks; the symptoms gradually disappeared, the pulse becoming natural, the pupils of the natural size, the head and chest relieved, and the bowels daily but fully moved; quiet sleep, and a good appetite returned.

In six days the patient was convalescent; shortly afterwards she bore a long journey home without any ill consequence, and at the proper time, had a safe delivery." 225.

Those forms of this morbid affection, says our author, which resemble peritonitis and pleuritis, are equally characterized by alternate chill or rigor and heat—frequency of pulse—and susceptibility to the effects of loss of blood. They do not occur so frequently or distinctly as the affection of the head. The following is a graphic description of this affection in general terms.

"It generally begins in the manner of a sudden attack. This attack is usually ushered in by rigor, indeed by a more distinct and decided rigor than is observed in many cases of inflammation; the rigor is usually soon followed by much heat of surface; with the heat the patient experiences some affection of the head, chest, or abdomen, and, indeed, frequently of all; there are vertigo on raising the head, pain, and some morbid impression on the mind, panting in the breathing, fluttering about the heart, with general hurry, irritability, and restlessness; the tongue is white and loaded; the alvine evacuations are morbid,—dark-coloured, fœtid, and scybalous,—or yellow like the yolk of egg,—or of the appearance of yeast; the urine is turbid and frequently deposits a copious sediment.

The affection of the head consists of the most acute pain, the greatest intolerance of light and sound, and the severest form of vertigo, wakefulness, and distress, and sometimes even delirium, and the pupils of the eyes are often extremely contracted.

The affection of the chest is denoted by severe and acute local pain, which is apt to vary its situation, passing from one side to the other, or to the back, or occupying a situation higher up or lower down: this pain checks a deep inspiration, and even the ordinary breathing, to which it imparts a character of difficulty and anxiety.

When the abdomen is affected, there are acute pain, and great tenderness under pressure, in some part, or more or less generally diffused. The attack and situation of the pain is such, in some instances, that the case is with difficulty distinguished from gall stones, though it more generally resembles peritonitis.

When the heart is the seat of this affection, there are violent and terrific attacks of palpitation, and the course of the carotids, and even of the abdominal aorta, is sometimes the seat of violent pulsation or throbbing.

All these affections are apt to occur in sudden attacks, and to recur in paroxysms,—perhaps varying their form,—and exciting great alarm in the patient and his friends, who usually dispatch a hurrying message to the medical attendants." 232.

The cases and observation which have been laid down establish the fact, that there are attacks which resemble inflammation of the head, chest, or abdomen, and yet are totally different in their nature—a fact that is highly important, in regard to diagnosis, on which the well-being of the case depends. The following diagnostic marks cannot be abridged without detriment.

" I would first observe that the attack of irritation, is, in general, more sudden than that of inflammation, which is usually formed somewhat more gradually. This circumstance must therefore be cautiously inquired into, and may assist the diagnosis.

I believe, too, that the seizure in the former case is attended by more distinct rigor, and afterwards by greater heat, than in the latter.

The case of irritation affects, in a marked degree, more organs at once, than that of inflammation, which is usually confined, at first at least, to one.

The state of the tongue and the condition of the alvine evacuations are far more marked by disorder, and the latter are far more offensive, in attacks from irritation than in cases of inflammation.

The affection of the head from irritation comes on suddenly, is formed all at once, and is frequently attended by great restlessness, suffering, and distress, and there is early syncope on taking blood. In arachnitis, the disease is usually formed somewhat more gradually; the patient has been subject to pain of the head perhaps for some days or even longer; he complains less; or at least there is less urgent distress,—less distress of a general kind; the pain may be very severe, although it is more frequently rather obscure; the intolerance of light and sound is less urgent; the rigor, and subsequent heat, and the attack in general are less marked; the patient is not so soon relieved by remedies, and the tongue and alvine evacuations are less morbid, and there is, especially, great tolerance of loss of blood. In the attack of affection of the head from irritation, the patient is relieved perhaps completely if the lancet be employed, but the attack soon recurs with equal or greater violence; in arachnitis, the relief is seldom so complete, the interval of ease so long, or the return so marked; the pain is diminished, perhaps, but gradually resumes its former violence, unless active measures be interposed.

When the chest is affected from irritation, the pain is severe and acute, and perhaps increased by a full inspiration; if the inspiration be repeated, however, a second and a third time, the increase of the pain is less and less; the situation of the pain varies; there is no cough, no crepitus on making a full expiration. In all these respects the case differs from inflammation. The remarks already made respecting the relief from remedies, the effect of bloodletting, the tendency to a sudden recurrence of the pain, &c. in cases of affection of the head, apply equally here.

I had long remarked that there might be both acute pain and tenderness under pressure, of the abdomen, without inflammation; this state of things is frequently the result of intestinal irritation. It is distinguished from inflammation by the general symptoms of this affection, the mode of attack, the effects of remedies. In inflammation, the surface is usually cool, the head unaffected, the patient remarkably quiet; in the case of irritation, on the contrary, there is generally much heat after rigor, the head is much affected, and the patient is restless and generally distressed, the tongue loaded and perhaps swollen, the alvine evacuations extremely morbid, and great relief is obtained by the free operation of medicine." 236.

Besides the circumstances already pointed out, Dr. H. draws the attention of the profession to some other points, which he considers as very interesting in their nature, and worthy of serious consideration. The *first* is, the occurrence of severe affection immediately consequent upon causes apparently inadequate to the production of such effects. Thus a slight blow, or a trifling fall, has appeared to produce an alarming indisposition.

" The truth is that there was already a disordered and loaded state of the bowels,—dormant until roused into effect by the accident. A lady about 50 years of age, fell a few steps down stairs; she got up however and walked to the sofa; in a short time she was taken with chilliness, succeeded by heat of skin and the most intolerable pain of the head and sensibility to light, noise, &c.

She soon recovered on taking active purges alternated with the ammoniacal anodyne draught." 237.

2ndly. Observant practitioners must have often remarked cases of apparent inflammation, probably yielding sooner than usual, or receding altogether, and recurring periodically. 3rdly. Such cases are sometimes relieved, but often refuse to yield to the lancet, recurring with great violence, when quite unexpected. 4thly. There is metastasis apparently of the local inflammation, whilst, in fact, the cause of the whole remained unremoved. 5thly. On dissection of apparent inflammations of internal organs no trace of the disease can be discovered. "The view which has been given of the effects of intestinal irritation may assist us in explaining an event which must have been witnessed by all who have, in any degree, pursued the study of morbid anatomy."

The mode of treatment consists in the free evacuation of the stomach and bowels—anodynes—light nourishment—and some local remedies. On each of these measures our author makes some judicious remarks; but these need not detain us longer, as their application is sufficiently obvious. He justly observes that, if our diagnosis could always be depended upon, there would probably be no occasion ever to use the lancet in such cases. But as, "judicium difficile," it is best, perhaps, to have cautious recourse to blood-letting even in intestinal irritation—since irritation may lead to inflammation.

The last two Chapters we must pass over, partly for want of space, but principally because their contents have been a good deal anticipated in former publications by the same author, and in previous numbers of this Journal. We have adduced sufficient proofs that Dr. Hall does not slacken in his pace of indefatigable observation, and unquenchable zeal, though we certainly regret that he does not give his works a more concentrated and systematic form; since they are now becoming so blended and reiterated that it requires a good memory to distinguish the old from the new—a keen taste to distinguish the meat fresh off the spit from that which has been hashed, even a second or third time. One thing, however, may be safely asserted, viz. that, whatever kind of viands Dr. Hall spreads upon the table of his guests, is wholesome and good—a recommendation of no mean value in the present day, when it cannot be said, with truth, that, "what does not poison will fatten."

